# Interleukin-7, but Not Thymic Stromal Lymphopoietin, Plays a Key Role in the T Cell Response to Influenza A Virus

**DOI:** 10.1371/journal.pone.0050199

**Published:** 2012-11-26

**Authors:** Adam W. Plumb, Daniel T. Patton, Jung Hee Seo, Emma-Kate Loveday, François Jean, Steven F. Ziegler, Ninan Abraham

**Affiliations:** 1 Infection, Inflammation and Immunity Research Group, Department of Microbiology & Immunology, Life Sciences Institute, University of British Columbia, Vancouver, British Columbia, Canada; 2 Department of Zoology, Life Sciences Institute, University of British Columbia, Vancouver, British Columbia, Canada; 3 Immunology Program, Benaroya Research Institute, Seattle, Washington, United States of America; INSERM, France

## Abstract

The immune response to viral infection is ideally rapid and specific, resulting in viral clearance and establishment of immune memory. Some viruses such as HIV can evade such responses leading to chronic infection, while others like Influenza A can elicit a severe inflammatory response with immune-related complications including death. Cytokines play a major role in shaping the appropriate outcomes to infection. While Interleukin-7 (IL-7) has a critical role in T and B cell development, treatment with IL-7 has recently been shown to aid the adaptive T cell response in clearance of chronic viral infection. In contrast, the IL-7-related cytokine thymic stromal lymphopoietin (TSLP) has a limited role in lymphocyte development but is important in the immune response to parasitic worms and allergens. The role for these cytokines in the immune response to an acute viral infection is unclear. IL-7 and TSLP share IL-7Rα as part of their heterodimeric receptors with the gamma common chain (γc) and TSLPR, respectively. We investigated the role of IL-7 and TSLP in the primary immune response to influenza A infection using hypomorphic IL-7Rα (IL-7Rα^449F^) and TSLPR^−/−^ mice. We found that IL-7, but not TSLP, plays an important role in control of influenza A virus. We also showed that IL-7 signaling was necessary for the generation of a robust influenza A-specific CD4 and CD8 T cell response and that this requirement is intrinsic to CD8 T cells. These findings demonstrate a significant role for IL-7 during acute viral infection.

## Introduction

Influenza A virus is a common human pathogen which causes significant morbidity and mortality worldwide [1]. Seasonal strains that cause annual epidemics can cause severe disease in immunocompromised individuals, including the young and the elderly [2], [3]. Novel reassortments of viral genes can occasionally result in highly pathogenic strains with the potential to cause severe disease in healthy adults [4]–[6]. Although a robust immune response is necessary to clear the virus, highly pathogenic strains can induce an overactive response that can significantly contribute to disease. Thus, a more detailed understanding of the determinants of the outcome of response to Influenza A infection might assist in preventative or therapeutic approaches.

Influenza A/PR/8/34 (H1N1) (referred herein as “PR8”) was derived from a human influenza strain and subsequently adapted to transmit from ferrets to mice [7]. This influenza A strain infects lung epithelial cells, forming a local infection that resolves in 10 days in wild-type mice [8]. Resolution of the primary infection is dependent on development of a well-defined influenza A specific T cell response. As a result, it is a clinically relevant model of a local respiratory virus infection.

Interleukin-7 (IL-7) has a central role in the development of the adaptive immune system and its response [9], [10]. While IL-7 has been primarily characterized for its role in lymphocyte development, it has also been recently shown to boost the T cell response against chronic viral infections and tumors [11], [12]. The role of the IL-7-related cytokine thymic stromal lymphopoietin (TSLP) has been more recently appreciated in the development of allergic and anti-parasite responses [13]. However, the role of these cytokines in acute viral infection is not clear.

IL-7 exerts it effects via a heterodimeric receptor of IL-7Rα paired with the gamma common chain (γc) [14]. IL-7Rα also serves as a receptor for TSLP when it is paired with TSLPR [15]. IL-7Rα and TSLPR are expressed on T cells and dendritic cells, as well as other innate immune cells with most of these cells co-expressing the γc. IL-7 is currently in clinical trials to enhance the response of and expand T cells in patients infected with HIV as well as in cancer patients and after bone marrow transplants [16].

TSLP acts primarily in the generation of immune response against parasites, however, in contrast to IL-7, it has little role in the development of the immune system [17]. TSLP has effects on the adaptive immune system both directly on T cells and via innate immune cells, such as dendritic cells [18]–[20]. TSLP is over expressed in many atopic diseases in humans including asthma and dermatitis [21]. Its expression can also be induced in bronchial epithelial cells by viral molecular motifs, such as CpG, suggesting it may play a role in defense against viral infections [22].

Signaling downstream of IL-7Rα is largely dependent on phosphorylation of tyrosine 449, nested within a YxxM recruitment motif. Mice bearing a mutated receptor with homozygous knock-in replacement of this tyrosine with phenylalanine (IL-7Rα^449F^) have impaired signaling in response to IL-7 [23]. Although IL-7Rα^449F^ mice have fewer mature T cells compared to WT mice, the lymphocyte compartment is significantly restored compared to IL-7Rα^−/−^ mice. This allows functional studies to be performed on these mice to investigate the role of IL-7Rα Tyr449 signaling. We showed that IL-7Rα^449F^ mice have a defective CD4 T cell response to *Listeria monocytogenes* infection, which suggested an unexpected role for IL-7-related cytokines in the primary T cell response [23]. Although IL-7 can boost T cell function during chronic inflammation, its role in the acute primary T cell response is unknown. Similarly, while TSLP has defined roles in the immune response to parasites, its role in viral infections has not been characterized.

We have investigated the role of IL-7 family cytokines during acute respiratory infection in mice infected with influenza A PR8. We found that IL-7, but not TSLP, plays an important role in viral clearance and protection from weight loss. We showed that IL-7 signaling in CD4 and CD8 T cells is critical for the development of the antigen-specific anti-influenza A response. These results highlight the role for IL-7 signaling in the antiviral effector T cell response and its potential to modulate the immune response to respiratory viruses.

## Materials and Methods

### Ethics Statement

Mice were housed at the UBC Centre for Disease Modelling facility, and all work was carried out according to University of British Columbia Animal Care and Biosafety Committee guidelines. Breeding and project protocols (A07–0415 and A07–0417) were approved by the institutional committee for this work. All efforts were made to minimize suffering, with minimally invasive procedures, and where warranted (viral infection), isoflurane anesthesia was used.

### Mice

C57BL/6, BoyJ (B6.SJL-*Ptprc^a^ Pepc^b^*/BoyJ) and Rag1^−/−^ (B6.129S7-*Rag1* ^tm1Mom^/J) mice were obtained from the Jackson Laboratory (Bar Harbour, MA). IL-7Rα^449F^ mice have a mutant form of the IL-7Rα expressing a single amino acid mutation from Tyr to Phe at position 449 as previously described [23]. TSLPR^−/−^ mice [17] were a gift of Dr. James Ihle (St. Jude Children’s Research Hospital, Memphis, TN). All mouse strains have been backcrossed at least ten times with C57BL/6 mice. Age- and sex-matched mice between 6 and 9 weeks of age were used for all experiments.

### Infection

Influenza A/PR/8/34 (PR8) was obtained from Charles River Laboratories (Wilmington, MA). Mice were anaesthetized using isoflurane and infected intranasally with 5 hemagglutinin units (HAU) of PR8 in 12.5 µL of sterile PBS.

### Cell Preparation and Flow Cytometry

Broncheoalveolar lavage (BAL) fluid was obtained by inserting a tracheal catheter and washing the bronchoalveolar space four times with 1 mL of PBS with 10% FBS. Lymphocytes were extracted from the lungs of mice by mincing with scissors, digesting with 100 units/mL Collagenase IV for 1 hour at 37°C before filtering with a 70 µm filter to remove debris. Spleens were collected and forced through a 70 µm filter to obtain a single cell suspension.

Antibodies were purchased from BD Bioscience (San Diego, CA), eBioscience (San Diego, CA) or BioLegend (San Diego, CA). H2-K^b^ and I-A^b^ tetramers loaded with immunodominant NP_366–374,_ PA_224–233_ and NP_311–325_ peptides from influenza A and labeled with PE or APC were made by the NIH Tetramer Core Facility (Atlanta, GA).

Tetramer staining was carried out at RT for 10 minutes before antibody staining at 4°C for 30 min. Data was acquired on a LSRII using FacsDiva software (BD Bioscience) and analysed using FlowJo software (TreeStar, Stanford, CA).

### Plaque Assays

Lungs from infected mice were homogenized using a Fisher Tissuemiser, diluted and incubated on confluent MDCK cells for 1 hour at RT. The cells were then washed and a solution of 0.7% agarose, 0.1% trypsin in α-MEM was applied. Samples were incubated at 37°C for 4 days before staining with crystal violet and counting of plaques.

### M1 RNA Detection

Lung from infected mice were homogenized in Trizol (Invitrogen), RNA was extracted as per manufacturer’s instructions and cDNA was prepared. Levels of viral Matrix gene transcript were then detected using M1F AGATGAGTCTTCTAACCGAGGTCG, M1R TGCAAAAACATCTTCAAGTCTCTG and an internal probe TCAGGCCCCCGCAAAGCCGA in duplicate using a Mx3005p PCR multiplex quantitative PCR instrument (Stratagene) with the Qiagen Quantitect qRT-PCR Kit (Qiagen). Total M1 copy numbers were determined by comparison to *in vitro* transcribed standards of known concentration [24].

### Mixed Bone Marrow Chimeras

CD45.1 mice were irradiated with two doses of 6.5 Gy four hours apart and then injected 24 hours later with a 9∶1 mixture of IL-7Rα^449F^:BoyJ or 1∶1 mixture of TSLPR^−/−^:BoyJ whole bone-marrow. Mice were infected with influenza A virus as above 6–8 weeks later.

### Statistical Analysis

Data are presented as mean±SEM and analyzed by Student’s t test or one-way ANOVA with Tukey’s post-test as appropriate. Results giving a p-value of less than 0.05 were considered to be significant.

## Results

### IL-7 is Critical for Control of and Protection from Influenza A Virus Infection

**Figure 1:**
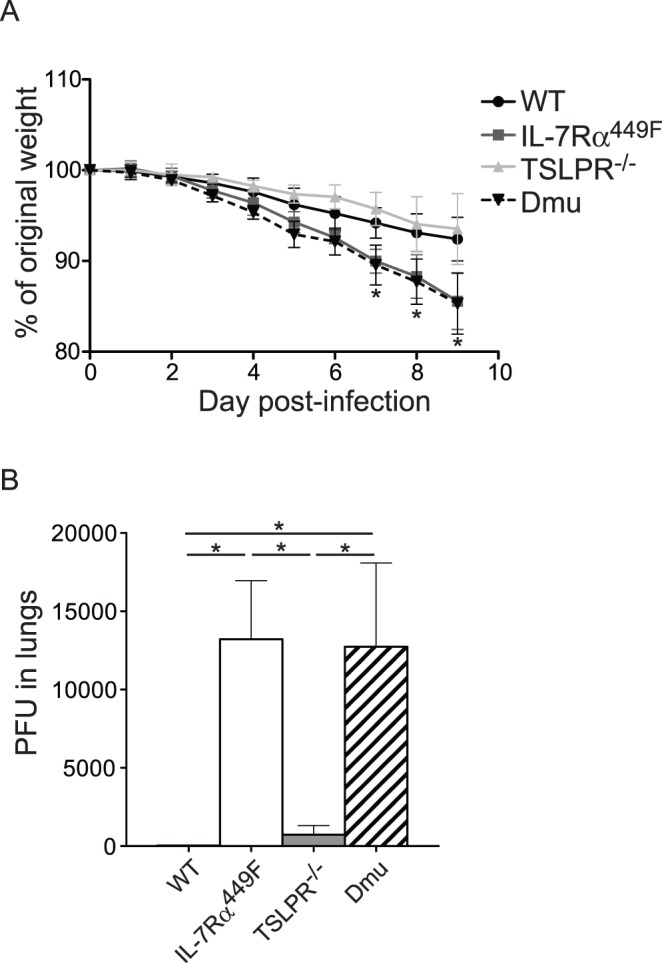
IL-7Rα signaling is required for control of Influenza A virus. A) C57BL/6, IL-7Rα^449F^, TSLPR^−/−^ and Dmu mice were infected intranasally with 5 HAU of Influenza A PR8. Mice were weighed daily, and the mean change in body mass plotted. Weight is expressed as a percentage of the weight on day 0. B) Lungs from mice at day 9 post infection were homogenized and Influenza A virus was measured by plaque assay. Data shown is from one representative experiment of three experiments, n = 3–5 *p<0.05 by ANOVA with Tukey’s post-test.

To assess the role of IL-7Rα signaling in influenza A pathogenicity, WT, IL-7Rα^449F^, TSLPR^−/−^ and IL-7Rα^449F^ TSLPR^−/−^ double-mutant (referred herein as “Dmu”) mice were infected with 5 HAU of influenza A PR8 by intranasal inoculation. This complement of genetic strains allowed us to distinguish the requirement for IL-7 or TSLP. We monitored mice for weight loss as an indicator of the severity of disease. While TSLPR^−/−^ mice showed similar weight loss compared to WT mice, both IL-7Rα^449F^ and Dmu mice lost significantly more weight from day 7 post-infection onwards compared to WT (Fig. 1a) suggesting that IL-7, not TSLP, was critical for protection from influenza A. We then determined whether the weight loss seen was due to a failure to clear the virus by examining the levels of infectious virus in the lungs of mice nine days after infection. While WT lung homogenates did not develop detectable plaques on MDCK cells and TSLPR^−/−^ homogenates had low numbers of plaques, the lung homogenates from IL-7Rα^449F^ and Dmu mice had elevated levels of infectious virus (Fig. 1b). These results suggested that while TSLP had no role in control of influenza A virus or protection from virus induced weight loss, IL-7 signaling through IL-7Rα^Y449^ was critical for both. Interestingly, viral M1 transcript levels were only elevated in Dmu mice (Figure S1), showing a discrepancy between the level of detectable infectious virus and viral transcripts in IL-7Rα^449F^ mice for unknown reasons.

### IL-7 Signaling is Necessary for a Robust Specific T Cell Response against Influenza A Virus

**Figure 2:**
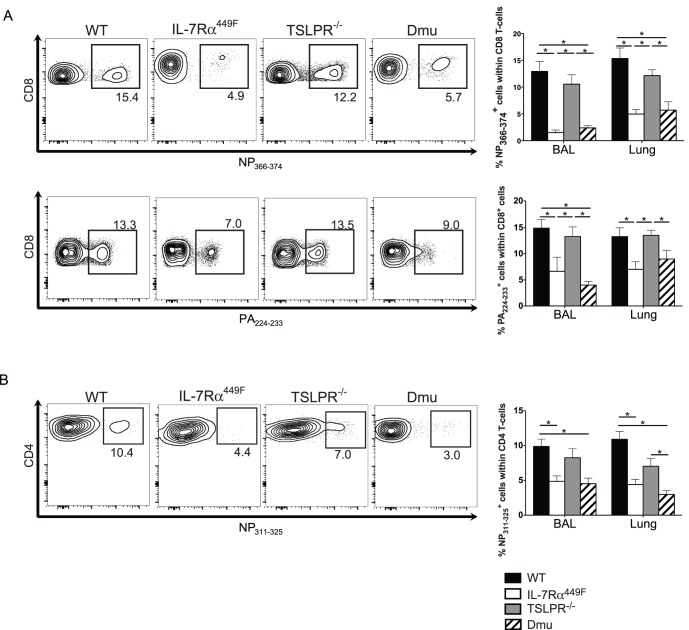
CD4 and CD8 T cell response to Influenza A requires IL-7Rα, but not TSLPR, signaling. A) Representative FACS plots and bar graphs of percent of total CD8 T cells specific for NP_366–374_ (top) or PA_224–233_ tetramer (bottom) in the BAL and lung. B) Representative FACS plots and bar graph of percent of total CD4 T cells specific for NP_311–325_ tetramer in the BAL and lung. Data shown is from one representative experiment of three experiments, n = 3–5. *p<0.05 by ANOVA with Tukey’s post-test.

As IL-7Rα signaling is necessary for the formation of an effective primary CD4 T cell response and influences the response to TCR stimulation [23], we examined whether IL-7Rα was important for the formation of the T cell response to influenza A. We evaluated the CD4 and CD8 T cell response in WT, IL-7Rα^449F^, TSLPR^−/−^ and Dmu mice infected with influenza A virus. The number of CD4 and CD8 T cells in the lung and BAL of IL-7Rα^449F^ and Dmu mice was reduced compared to WT after infection, while TSLPR^−/−^ mice had normal numbers of CD4 and CD8 T cells (Fig. S2). However, as both IL-7Rα^449F^ and Dmu mice have reduced number of baseline CD4 and CD8 T cells in the spleen and lung prior to infection (Figure S3) we examined the frequency of CD8 T cells that recognize the virus with MHC class I tetramers containing immunodominant viral epitopes NP_366–374_ and PA_224–233_ [25], [26]. At day 9 post-infection, the proportion of CD8 T cells that recognized both the NP_366–374_ and PA_224–233_ tetramers was significantly reduced in IL-7Rα^449F^ compared to WT in both the BAL and the lung (Fig. 2a). IL-7Rα^449F^ mice also had reduced frequency of CD4 T cells that recognize the NP_311–325_ epitope (Fig. 2b). In contrast, TSLPR^−/−^ mice had similar levels of tetramer-binding CD4 and CD8 T cells compared to WT mice and Dmu mice had a comparable T cell response to IL-7Rα^449F^ mice. These results indicate that IL-7, but not TSLP, was important for the influenza A specific T cell response.

### IL-7 and TSLP Regulate CXCR3 Expression on Virus Specific T Cells

**Figure 3:**
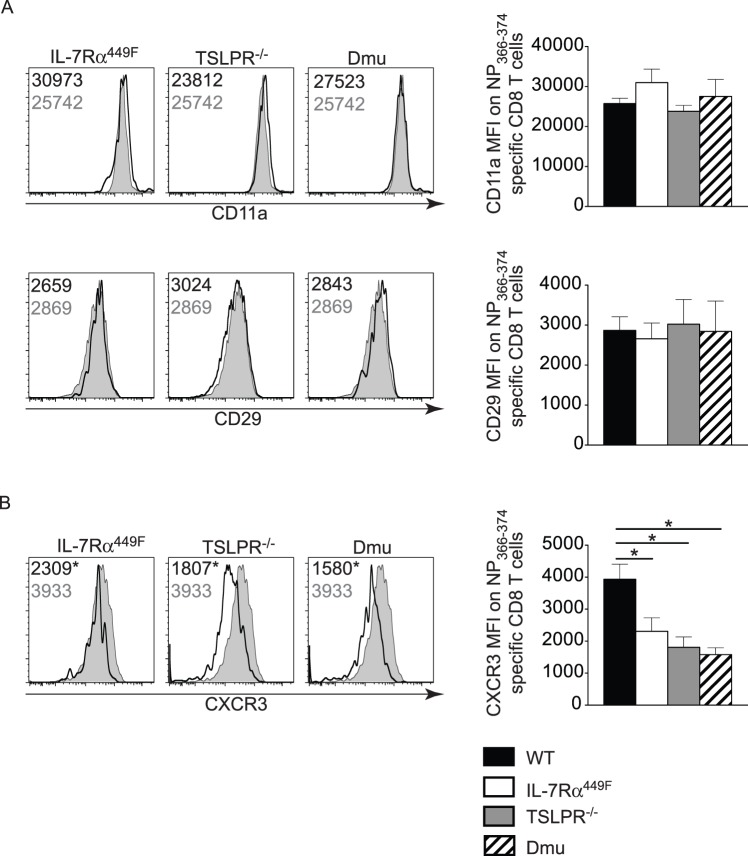
Expression of CD11a, CD29 and CXCR3 by Influenza A specific T cells. Lungs from mice were taken nine days after infection and stained for CD11a, CD29 and CXCR3. Events shown were gated on NP_366–374_ tetramer^+^ CD8 T cells. Grey histograms are a representative WT control, black lines are from the labeled mutant strain. The mean fluorescence intensity of each marker is shown on the representative FACS plots and bar graph. One representative experiment of two experiments with n = 3–5. *p<0.05 by ANOVA with Tukey’s post-test.

We then asked if the reduction in T cells seen in the lung of infected IL-7Rα^449F^ mice was due to a failure of cells to migrate from the lymph nodes into the infected areas of the lung and airways. Several integrins and chemokine receptors, in particular CD11a [27], CD29, and CXCR3 [28] have been implicated in migration of T cells to the lung during influenza A infection. Although IL-7 has been reported to influence expression of the integrins CD11a and CD29 [29], we found no difference in the expression of these proteins on influenza specific T cells from IL-7Rα^449F^, TSLPR^−/−^ or Dmu mice (Fig. 3a). However, there was a significant reduction in the expression of the chemokine receptor CXCR3 on influenza A specific CD8 T cells from the lungs of infected IL-7Rα^449F^, TSLPR^−/−^ and Dmu mice. This suggested that IL-7 and TSLP may both regulate the expression of CXCR3 in mice infected with influenza A.

### Generation of Influenza A Specific T Cells Requires Cell Intrinsic IL-7 Signaling

**Figure 4:**
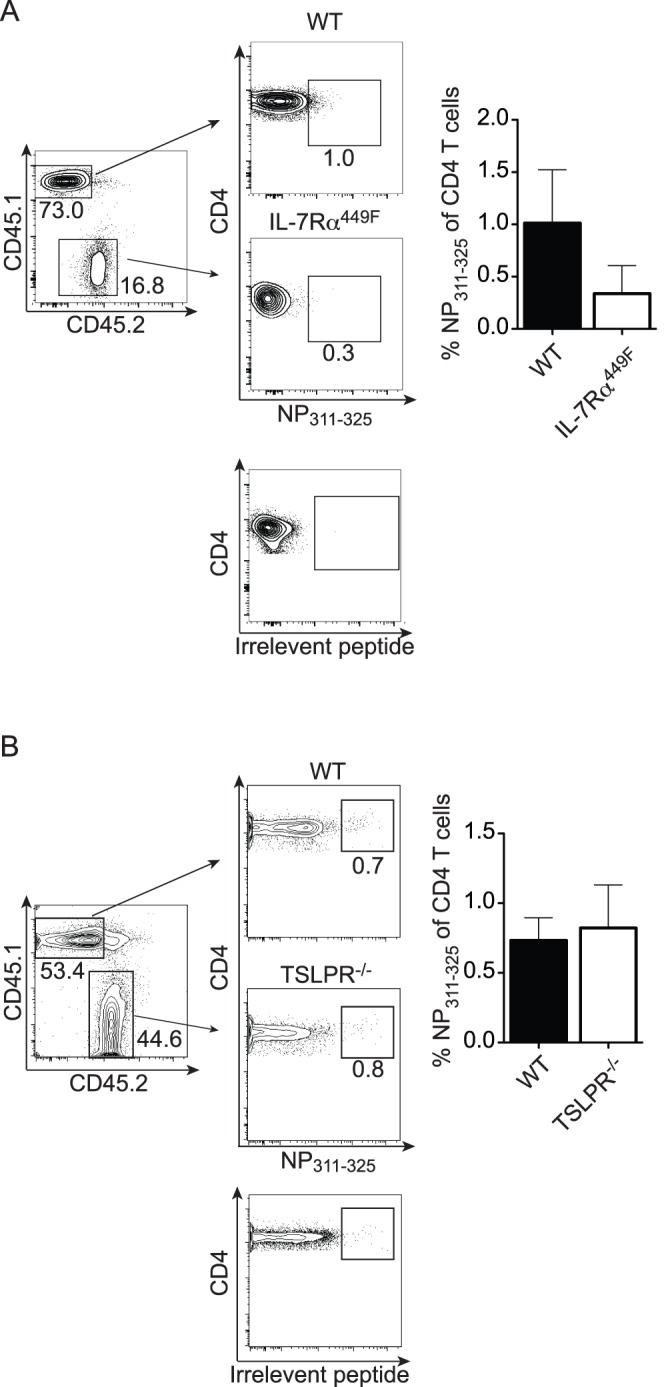
Cell-intrinsic requirement for IL-7Rα, but not TSLPR, signaling in CD8 T cell response to Influenza A. Lethally irradiated CD45.1 BoyJ mice were reconstituted with (A) 9∶1 IL-7Rα^449F^:BoyJ or (B) 1∶1 TSLPR^−/−^:BoyJ bone-marrow and allowed to recover for 6–8 weeks. Mice were infected with 5HAU of PR8 and the T cell response was analyzed at day 9 post-infection. CD8 T cells were stained for CD45.1 and CD45.2 to identify WT or mutant derived cells. Each population was analyzed for the percent of total CD8 T cells that recognize the NP_366–374_ tetramer. Data shown is from one representative experiment of two experiments, n = 3–7. *p<0.05 by Student’s t-test.

To investigate whether the requirement for IL-7Rα signaling was due to signaling within T cells, we made a series of mixed bone marrow chimeras to examine the response of congenically marked WT and mutant lymphoid cells within the same mouse. Irradiated RAG1^−/−^ mice were reconstituted with a mixture of BoyJ (WT, CD45.1^+^) and IL-7Rα^449F^ (CD45.2^+^) bone marrow. As IL-7Rα^449F^ T cells performed poorly in competitive repopulation against WT during development [23] we transferred bone marrow at a ratio of 9∶1 IL-7Rα^449F^:WT to obtain mice with a sufficiently large IL-7Rα^449F^ CD8^+^ T cell compartment to accurately evaluate their response. Reconstituted mice were then infected with influenza A six weeks after irradiation and transplantation and the frequency of influenza A specific CD8 T cells within either the WT or IL-7Rα^449F^ derived cells was examined at day 9 post-infection. As expected, IL-7Rα^449F^ T cells were heavily outcompeted by WT T cells during development (Fig. 4a). As a result, IL-7Rα^449F^ T cells make up only 20% of the total T cell population in these mice, despite having been reconstituted at a 9∶1 advantage. This output ratio is consistent with chimerism we have observed in uninfected mice (data not shown). Our data showed that within the same host, the frequency of NP_366–374_ specific cells among CD8 T cells in the lung was significantly reduced in the IL-7Rα^449F^ population compared to the WT population (Fig. 4a). The NP_311–325_ CD4 T cell response in IL-7Rα^449F^ cells also showed a trend towards impairment, but this was not a significant difference (Figure S4), perhaps due to the lack of a robust response in WT CD4 T cells in these chimeric mice. Taken together, our data clearly indicated a cell-intrinsic requirement for IL-7Rα signaling in the development of the specific CD8 T cell response to influenza A.

TSLP has been shown to influence CD8 T cell homeostasis and survival in competition with WT cells although no defect was observed in TSLPR^−/−^ mice [30]. To determine if a role for TSLP in the generation of influenza A specific T cells could be observed in competition with WT cells, we transferred a 1∶1 mixture of TSLPR^−/−^:WT bone marrow into irradiated recipients. Reconstituted mice were then infected with influenza A six weeks after irradiation and transplantation, and the frequency of influenza A specific CD8 T cells within either the WT or TSLPR^−/−^ derived cells was examined at day 9 post-infection. WT and TSLPR^−/−^ T cells contributed equally to the influenza A specific CD8 T cell populations in the lung (Fig. 4b) and BAL (data not shown). Furthermore, the NP_311–325_ specific CD4 T cell response in the TSLPR^−/−^ compartment also showed no impairment, consistent with direct infection of TSLPR^−/−^ mice (Figure S4). Our data refute a role for cell intrinsic TSLP signaling in the T cell response to influenza A, suggesting that the observed defects in IL-7Rα^449F^ cells in chimeric animals were due to the loss of IL-7 signals.

## Discussion

IL-7 and TSLP have well described roles as modulators of T cell immunity. IL-7 has been shown to enhance the T cell response against chronic viral infections and tumours [11], [12] and we have previously reported its role in the T cell response to systemic challenge by *Listeria monocytogenes*. TSLP has primarily been characterized for its role in Th2-mediated immune responses, however it can be strongly induced by a variety of viral ligands and has been shown to influence the T cell response to BCG [22], [31]. The role of IL-7 in mature lymphocytes is difficult to study due to the severity of B and T cell defects in IL-7Rα^−/−^ mice [32]. We previously generated IL-7Rα^449F^ mice that are hypomorphic for IL-7Rα function. Unlike IL-7Rα^−/−^ mice, these mice develop mature B and T cells allowing evaluation of the role of IL-7Rα signaling in mature lymphocyte function. By comparing IL-7Rα^449F^ mice to WT and TSLPR^−/−^ mice we are able to identify discrete roles for IL-7 or TSLP for an observed phenotype.

We showed that IL-7-mediated signaling was necessary for the generation of the primary CD4 and CD8 T cell responses against a physiologically relevant, acute influenza A mucosal challenge. The impaired CD4 T cell response to influenza A in IL-7Rα^449F^ mice is consistent with our previous findings with a systemic *Listeria monocytogenes* challenge [23]. However, the defect in the primary CD8 T cell response in IL-7Rα^449F^ mice to influenza A was surprising as no such defect was observed in the *Listeria* study. As both CD4 and CD8 IL-7Rα^449F^ T cells showed a dose dependent defect in anti-CD3 induced division [23], we postulate the route of infection and antigenicity of pathogen challenge can determine the requirement for IL-7R signals. Our current model is that TCR engagement by sub-optimal antigenic stimuli is enhanced by IL-7R signals, is impaired in IL-7Rα^449F^ T cells and that this can be negated by strong T cell receptor signaling.

Our data showed no role for TSLP in the CD8 T cell response to influenza A PR8 in contrast to recent findings [33]. As alterations in the gut normal flora can influence the immune response to influenza A [34], it is possible that differences in the flora of the mice alter the contributions of the TSLP dependent response in these mice. Alternatively, although both studies examined low dose challenges of influenza A, the virus dose may still contribute to the differences seen between the studies.

Previous studies have shown that depletion of lymphoid subsets could raise the susceptibility of mice to influenza A infection [8]. However, these studies were performed in mice that lacked one or more lymphoid subsets. Models of partial lymphopenia have not been studied and thus it is unclear whether the reduced number of CD4 and CD8 T cells in IL-7Rα^449F^ mice contribute to the increased severity of the influenza A infection in these mice. Nonetheless, our bone marrow chimeras with IL-7Rα^449F^ T cells clearly showed in lymphoreplete mice that IL-7Rα signals are necessary for an effective T cell response. Thus, while lymphopenia may contribute to disease severity in IL-7Rα^449F^ mice, it is by no means the sole reason for the loss of viral control observed.

Migration of cells to the site of influenza infection is dependent on expression of integrins and chemokine receptors. We found no defect in the expression of the integrin subunits CD11a or CD29. IL-7Rα^449F^ and TSLPR^−/−^ mice both showed reduced expression of CXCR3. However due to the lack of a defect in both the proportion of tetramer positive cells or disease in TSLPR^−/−^ mice, we would argue that although interesting, this chemokine receptor is not required for the T cell response to influenza A. The contribution of a migratory defect to the T cell deficiency observed in the lung could be determined by comparing the number of influenza A specific T cells in the lung versus the draining lymph nodes. If the response is normal in the mediastinal lymph node in IL-7Rα^449F^ mice, this would imply that defective migration of the cells to the lung, and not initiation and amplification of the T cell response is responsible for the reduction in influenza A specific T cells observed in these mice.

IL-7 has a clear role in survival and homeostasis of T cells, which is in part mediated by signaling through STAT5. STAT5 also has a critical role in maintaining the T cell response to LCMV as expression of dominant-negative STAT5 in T cells results in a reduction in cellular proliferation and survival [35]. IL-7Rα^449F^ cells do not phosphorylate STAT5 in response to IL-7, suggesting that the defect seen in this mouse may mirror that seen in dominant-negative STAT5 mutant cells.

We show that IL-7, and not TSLP, plays an important role in the control of influenza A virus and in the generation of the influenza A specific T cell response. These findings have implications for shaping therapies directed at use of IL-7 in immune reconstitution and inhibition of TSLP in atopic pathologies.

## Supporting Information

Figure S1 **Quantification of M1 viral RNA in the lungs.** C57BL/6, IL-7Rα^449F^, TSLPR^−/−^ and Dmu mice were infected intranasally with 5HAU of influenza A PR8. At day 9 post-infection lungs were harvested, homogenized in Trizol and absolute amount of influenza A M1 mRNA was determined by qPCR. Data shown is pooled from three experiments. *p<0.05 by ANOVA with Tukey’s post-test.
(EPS)

Figure S2 **Total CD4 and CD8 T cell response in the lung and BAL.** Total number of (A) CD8 and (B) CD4 T cells in the lung and BAL in day 9 post-infection from C57BL/6, IL-7Rα^449F^, TSLPR^−/−^ and Dmu mice. Data shown are from one representative experiment of three experiments, n = 3–5. *p<0.05 by ANOVA with Tukey’s post-test.
(EPS)

Figure S3 **Baseline CD4 and CD8 T cell in the lung and spleen.** Total number of (A) CD8 and (B) CD4 T cells in the lung and spleen from uninfected C57BL/6, IL-7Rα^449F^, TSLPR^−/−^ and Dmu mice. Data shown are from one representative experiment of three experiments, n = 3–4. *p<0.05 by ANOVA with Tukey’s post-test.
(EPS)

Figure S4 **Cell-intrinsic requirement for IL-7Rα, but not TSLPR, signaling in CD4 T cell response to Influenza A.** Lethally irradiated CD45.1 BoyJ mice were reconstituted with (A) 9∶1 IL-7Rα^449F^:BoyJ or (B) 1∶1 TSLPR^−/−^:BoyJ bone-marrow and allowed to recover for 6–8 weeks. Mice were infected with 5HAU of PR8 and the CD4 T cell response was analyzed at day 9 post-infection. CD4 T cells were stained for CD45.1 and CD45.2 to identify WT or mutant derived cells. Each population was analyzed for the percent of CD4 T cells that recognize the NP_311–325_ tetramer. Irrelevant tetramer staining on CD4 T cells is shown below NP_311–325_ staining in each panel. Data shown are from one representative experiment of two experiments, n = 3–7. *p<0.05 by Student’s t-test.
(EPS)

## References

[1] Bridges CB, Winquist AG, Fukuda K, Cox NJ, Singleton JA, et al. (2000) Prevention and control of influenza: recommendations of the Advisory Committee on Immunization Practices (ACIP). MMWR Recomm Rep 49: 1–38; quiz CE31–37. 15580733

[2] Prosser LA, Bridges CB, Uyeki TM, Hinrichsen VL, Meltzer MI, et al (2006) Health benefits, risks, and cost-effectiveness of influenza vaccination of children. Emerg Infect Dis 12: 1548–1558. 17176570 10.3201/eid1210.051015 PMC3290928

[3] Webster RG (2000) Immunity to influenza in the elderly. Vaccine 18: 1686–1689. 10689149 10.1016/s0264-410x(99)00507-1

[4] Dominguez-Cherit G, Lapinsky SE, Macias AE, Pinto R, Espinosa-Perez L, et al (2009) Critically Ill patients with 2009 influenza A(H1N1) in Mexico. JAMA 302: 1880–1887. 19822626 10.1001/jama.2009.1536

[5] Kumar A, Zarychanski R, Pinto R, Cook DJ, Marshall J, et al (2009) Critically ill patients with 2009 influenza A(H1N1) infection in Canada. JAMA 302: 1872–1879. 19822627 10.1001/jama.2009.1496

[6] Davies A, Jones D, Bailey M, Beca J, Bellomo R, et al (2009) Extracorporeal Membrane Oxygenation for 2009 Influenza A(H1N1) Acute Respiratory Distress Syndrome. JAMA 302: 1888–1895. 19822628 10.1001/jama.2009.1535

[7] Francis T Jr (1934) Transmission of Influenza by a Filterable Virus. Science 80: 457–459. 10.1126/science.80.2081.457-a 17795179

[8] Brown DM, Roman E, Swain SL (2004) CD4 T cell responses to influenza infection. Semin Immunol 16: 171–177. 15130501 10.1016/j.smim.2004.02.004

[9] Fewkes NM, Mackall CL (2010) Novel gamma-chain cytokines as candidate immune modulators in immune therapies for cancer. Cancer J 16: 392–398. 20693852 10.1097/PPO.0b013e3181eacbc4 PMC6959548

[10] Rochman Y, Spolski R, Leonard WJ (2009) New insights into the regulation of T cells by gamma(c) family cytokines. Nat Rev Immunol 9: 480–490. 19543225 10.1038/nri2580 PMC2814538

[11] Pellegrini M, Calzascia T, Elford AR, Shahinian A, Lin AE, et al (2009) Adjuvant IL-7 antagonizes multiple cellular and molecular inhibitory networks to enhance immunotherapies. Nat Med 15: 528–536. 19396174 10.1038/nm.1953

[12] Pellegrini M, Calzascia T, Toe JG, Preston SP, Lin AE, et al (2011) IL-7 engages multiple mechanisms to overcome chronic viral infection and limit organ pathology. Cell 144: 601–613. 21295337 10.1016/j.cell.2011.01.011

[13] Ziegler SF, Artis D (2010) Sensing the outside world: TSLP regulates barrier immunity. Nat Immunol 11: 289–293. 20300138 10.1038/ni.1852 PMC2924817

[14] Kondo M, Takeshita T, Higuchi M, Nakamura M, Sudo T, et al (1994) Functional participation of the IL-2 receptor gamma chain in IL-7 receptor complexes. Science 263: 1453–1454. 8128231 10.1126/science.8128231

[15] Park LS, Martin U, Garka K, Gliniak B, Di Santo JP, et al (2000) Cloning of the murine thymic stromal lymphopoietin (TSLP) receptor: Formation of a functional heteromeric complex requires interleukin 7 receptor. J Exp Med 192: 659–670. 10974032 10.1084/jem.192.5.659 PMC2193276

[16] Lundstrom W, Fewkes NM, Mackall CL (2012) IL-7 in human health and disease. Semin Immunol 24: 218–224. 22410365 10.1016/j.smim.2012.02.005 PMC3358500

[17] Carpino N, Thierfelder WE, Chang MS, Saris C, Turner SJ, et al (2004) Absence of an essential role for thymic stromal lymphopoietin receptor in murine B-cell development. Mol Cell Biol 24: 2584–2592. 14993294 10.1128/MCB.24.6.2584-2592.2004 PMC355866

[18] Akamatsu T, Watanabe N, Kido M, Saga K, Tanaka J, et al (2008) Human TSLP directly enhances expansion of CD8+ T cells. Clin Exp Immunol 154: 98–106. 18727630 10.1111/j.1365-2249.2008.03731.x PMC2561086

[19] Gilliet M, Soumelis V, Watanabe N, Hanabuchi S, Antonenko S, et al (2003) Human dendritic cells activated by TSLP and CD40L induce proallergic cytotoxic T cells. J Exp Med 197: 1059–1063. 12707303 10.1084/jem.20030240 PMC2193883

[20] Watanabe N, Hanabuchi S, Soumelis V, Yuan W, Ho S, et al (2004) Human thymic stromal lymphopoietin promotes dendritic cell-mediated CD4+ T cell homeostatic expansion. Nat Immunol 5: 426–434. 14991051 10.1038/ni1048

[21] Ying S, O’Connor B, Ratoff J, Meng Q, Mallett K, et al (2005) Thymic stromal lymphopoietin expression is increased in asthmatic airways and correlates with expression of Th2-attracting chemokines and disease severity. J Immunol 174: 8183–8190. 15944327 10.4049/jimmunol.174.12.8183

[22] Allakhverdi Z, Comeau MR, Jessup HK, Yoon BR, Brewer A, et al (2007) Thymic stromal lymphopoietin is released by human epithelial cells in response to microbes, trauma, or inflammation and potently activates mast cells. J Exp Med 204: 253–258. 17242164 10.1084/jem.20062211 PMC2118732

[23] Osborne LC, Dhanji S, Snow JW, Priatel JJ, Ma MC, et al (2007) Impaired CD8 T cell memory and CD4 T cell primary responses in IL-7R alpha mutant mice. J Exp Med 204: 619–631. 17325202 10.1084/jem.20061871 PMC2137912

[24] Loveday EK, Svinti V, Diederich S, Pasick J, Jean F (2012) Temporal- and strain-specific host microRNA molecular signatures associated with swine-origin H1N1 and avian-origin H7N7 influenza A virus infection. J Virol 86: 6109–6122. 22438559 10.1128/JVI.06892-11 PMC3372180

[25] Day EB, Zeng W, Doherty PC, Jackson DC, Kedzierska K, et al (2007) The context of epitope presentation can influence functional quality of recalled influenza A virus-specific memory CD8+ T cells. J Immunol 179: 2187–2194. 17675478 10.4049/jimmunol.179.4.2187

[26] Sedgmen BJ, Dawicki W, Gommerman JL, Pfeffer K, Watts TH (2006) LIGHT is dispensable for CD4+ and CD8+ T cell and antibody responses to influenza A virus in mice. Int Immunol 18: 797–806. 16569673 10.1093/intimm/dxl016

[27] Thatte J, Dabak V, Williams MB, Braciale TJ, Ley K (2003) LFA-1 is required for retention of effector CD8 T cells in mouse lungs. Blood 101: 4916–4922. 12623847 10.1182/blood-2002-10-3159

[28] Fadel SA, Bromley SK, Medoff BD, Luster AD (2008) CXCR3-deficiency protects influenza-infected CCR5-deficient mice from mortality. Eur J Immunol 38: 3376–3387. 19039768 10.1002/eji.200838628 PMC2749081

[29] Unsinger J, McGlynn M, Kasten KR, Hoekzema AS, Watanabe E, et al (2010) IL-7 promotes T cell viability, trafficking, and functionality and improves survival in sepsis. J Immunol 184: 3768–3779. 20200277 10.4049/jimmunol.0903151 PMC2914630

[30] Rochman Y, Leonard WJ (2008) The role of thymic stromal lymphopoietin in CD8+ T cell homeostasis. J Immunol 181: 7699–7705. 19017958 10.4049/jimmunol.181.11.7699 PMC2735224

[31] Sugimoto H, Ito T, Torii Y, Amuro H, Yokoi T, et al (2010) Thymic stromal lymphopoietin plays an adjuvant role in BCG-mediated CD8(+) cytotoxic T cell responses through dendritic cell activation. Clin Immunol 136: 205–216. 20471323 10.1016/j.clim.2010.04.006

[32] Peschon JJ, Morrissey PJ, Grabstein KH, Ramsdell FJ, Maraskovsky E, et al (1994) Early lymphocyte expansion is severely impaired in interleukin 7 receptor-deficient mice. J Exp Med 180: 1955–1960. 7964471 10.1084/jem.180.5.1955 PMC2191751

[33] Yadava K, Sichelstiel A, Luescher IF, Nicod LP, Harris NL, et al. (2012) TSLP promotes influenza-specific CD8+ T-cell responses by augmenting local inflammatory dendritic cell function. Mucosal Immunol. (Epub ahead of print) doi: 10.1038/mi.2012.50. 10.1038/mi.2012.50 PMC3534170 22806096

[34] Abt MC, Osborne LC, Monticelli LA, Doering TA, Alenghat T, et al (2012) Commensal bacteria calibrate the activation threshold of innate antiviral immunity. Immunity 37: 158–170. 22705104 10.1016/j.immuni.2012.04.011 PMC3679670

[35] Hand TW, Cui W, Jung YW, Sefik E, Joshi NS, et al (2010) Differential effects of STAT5 and PI3K/AKT signaling on effector and memory CD8 T-cell survival. Proc Natl Acad Sci U S A 107: 16601–16606. 20823247 10.1073/pnas.1003457107 PMC2944719

