# Correction: Interleukin-7, but Not Thymic Stromal Lymphopoietin, Plays a Key Role in the T Cell Response to Influenza A Virus

**DOI:** 10.1371/journal.pone.0169498

**Published:** 2017-01-03

**Authors:** The PLOS ONE Staff

Fig 4 is incorrectly replaced with Supplemental S4 Fig. The publisher apologizes for the error.

Please see the correct Fig 4 here.

**Fig 4:**
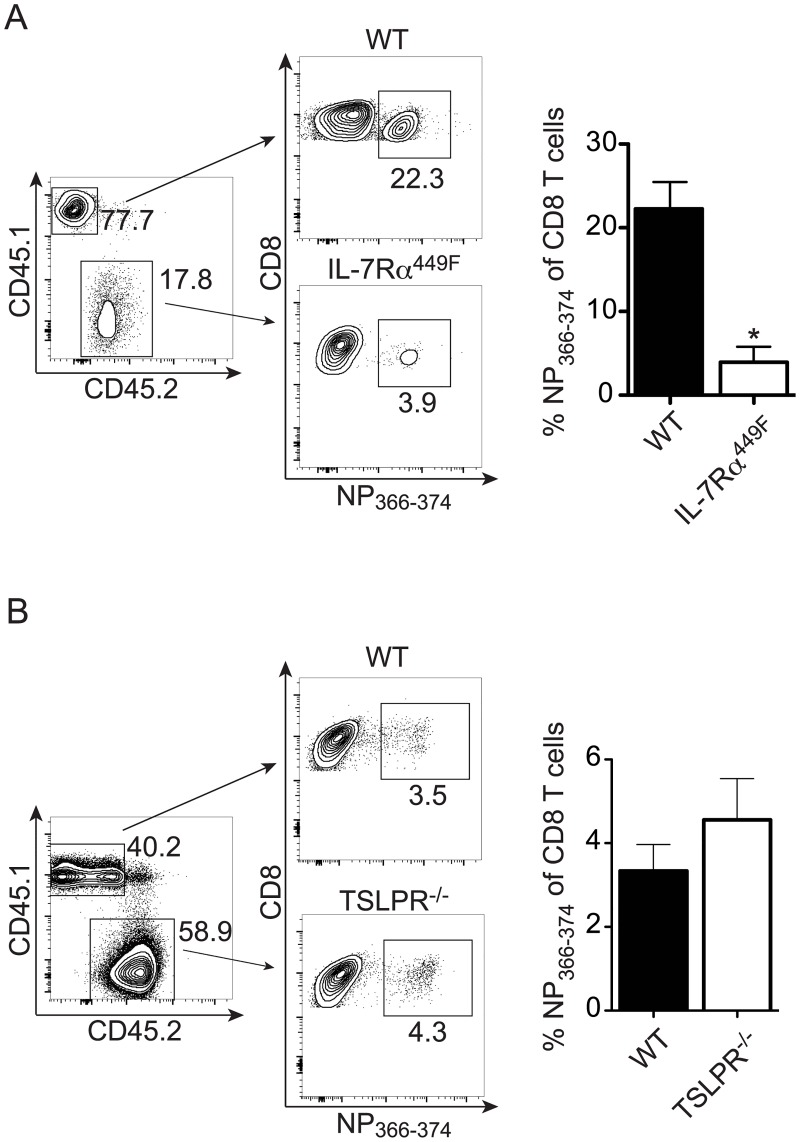
Cell-intrinsic requirement for IL-7Rα, but not TSLPR, signaling in CD8 T cell response to Influenza A. Lethally irradiated CD45.1 BoyJ mice were reconstituted with (A) 9:1 IL-7Rα^449F^:BoyJ or (B) 1:1 TSLPR^-/-^:BoyJ bone-marrow and allowed to recover for 6–8 weeks. Mice were infected with 5HAU of PR8 and the T cell response was analyzed at day 9 post-infection. CD8 T cells were stained for CD45.1 and CD45.2 to identify WT or mutant derived cells. Each population was analyzed for the percent of total CD8 T cells that recognize the NP_366–374_ tetramer. Data shown is from one representative experiment of two experiments, n = 3–7. *p<0.05 by Student’s t-test.

Supplemental S4 Fig has several errors. The Supplemental S4 Fig lacks changes made during production including the percent of subsets in various flow gates in Panels A and B. Furthermore, the x-axes’ labels titled “NP311-324” should read “NP311-325” in panels A and B. The publisher apologizes for the error.

Please see the correct Supplemental S4 Fig here.

## Supporting Information

S4 Fig Cell-intrinsic requirement for IL-7Rα, but not TSLPR, signaling in CD4 T cell response to Influenza A. Lethally irradiated CD45.1 BoyJ mice were reconstituted with (A) 9:1 IL-7Rα^449F^:BoyJ or (B) 1:1 TSLPR^-/-^:BoyJ bone-marrow and allowed to recover for 6–8 weeks. Mice were infected with 5HAU of PR8 and the CD4 T cell response was analyzed at day 9 post-infection. CD4 T cells were stained for CD45.1 and CD45.2 to identify WT or mutant derived cells. Each population was analyzed for the percent of CD4 T cells that recognize the NP_311–325_ tetramer. Irrelevant tetramer staining on CD4 T cells is shown below NP_311–325_ staining in each panel. Data shown is from one representative experiment of two experiments, n = 3–7. *p<0.05 by Student’s t-test.
(EPS)

## References

[1] Plumb AW, Patton DT, Seo JH, Loveday E-K, Jean F, Ziegler SF, et al (2012) Interleukin-7, but Not Thymic Stromal Lymphopoietin, Plays a Key Role in the T Cell Response to Influenza A Virus. PLoS ONE 7(11): e50199 doi: 10.1371/journal.pone.0050199 23189186 10.1371/journal.pone.0050199 PMC3506535

